# Study of Antiherpetic Efficiency of Phosphite of Acycloguanosine Ableto Over come the Barrier of Resistance to Acyclovir

**Published:** 2016

**Authors:** V. L. Andronova, M.V. Jasko, M.K. Kukhanova, G.A. Galegov, Yr.S. Skoblov, S.N. Kochetkov

**Affiliations:** D.I. Ivanovsky Institute of Virology (N.F. Gamaleya Research Center of Epidemiology and Microbiology, Ministry of Healthcare of the Russian Federation),Gamaleya Str., 16, Moscow, 123098 , Russia; V.A. Engelhardt Institute of Molecular Biology, Russian Academy of Sciences, Vavilova Str., 32, Moscow, 119991, Russia; M.M. Shemyakin-Yr.A.Ovchinnikov Institute of Bioorganic Chemistry, Russian Academy of Sciences, Miklukho-Maklaya Str., 16/10, Moscow, 117997, Russia

**Keywords:** herpes simplex virus, antiviral activity, in vitro, in vivo

## Abstract

As has been shown previously, phosphite of acycloguanosine (Hp-ACG) exhibits equal efficacy against ACV-sensitive and ACV-resistant HSV-1 strains in cell culture. Intraperitoneal administration of Hp-ACG to model mice with herpetic encephalitis caused by HSV-1 infection was shown to be effective in protecting against death. In the present work, we continue the study of the antiviral efficiency of Hp-ACG against HSV administered non-invasively; namely *in vivo*, orally and in the form of ointment formulations. It has been first shown that oral administration of Hp-ACG twice daily for five days prevents systemic infection in mice caused by HSV-1. Mortality in the control group of animals was 57%. Administration of Hp-ACG at doses of 600, 800 and 1,000 mg/kg per day significantly increased the survival and median day of death of the animals compared to the placebo-treated control group. A comparative evaluation of the therapeutic efficacy parameters of polyethylene glycol-based ACV ointment and Hp-ACG ointment was carried out after a 5-day course in the model of an experimental cutaneous infection of HSV-1 in guinea pigs. It was found that Hp-ACG has a significant therapeutic effect resulting in a statistically significant reduction in the lesion’s surface area and the amount of vesicular structures. The exhibited therapeutic effect of 10% Hp-ACG in ointment form compares well with that of 5% ACG ointment.

## INTRODUCTION

Infections caused by the herpes simplex virus (HSV) are widespread: from 50 to 95% of the world adult population possess antibodies to HSV-1, and 20–30% are carriers of HSV-2 by the age of 15–29 years, wherein the number of seropositive persons increases with age [1, 2]. After the initial infection, herpes viruses establish a lifelong latent infection with periodic recurrences. HSV can infect various organs and tissues, leading to a variety of clinical manifestations of the infection, including skin, the mucosas and eye lesions, and even generalized forms with damage to internal organs and CNS [3]. Therefore, it is important to have available dosage forms both for external use and systemic administration for the treatment of herpetic infections of various severities and localizations. For this purpose, nucleoside analogs are commonly used in medical practice. First of all, these are the known antiherpetic agents acyclovir (ACV, ACG, Zovirax, 9-[(2-hydroxyethoxy) methyl]-guanine) available either as 5% ointment, 5% cream or 3% ophthalmic ointment and in the form of tablets, 8% suspensions for internal use, lyophilized powder for infusion solution, and lyophilized powder for injection solution (GlaxoSmithKline group of companies and etc.). Moreover, first-line antiherpetic agents include penciclovir (PCV, 1% cream) and metabolic precursors of ACV and PCV: valine ester of ACV (Valtrex, tablets with a dosage of 250, 500, and 1000 mg) and famciclovir (Famvir, film-coated tablets, 125, 250, and 500 mg dosage forms).

However, herpes viruses develop resistance to these drugs. The frequency of isolation of HSV with drug resistance in clinical practice depends on the immune status and ranges from 0.5% in immunocompetent patients to 2–36% in immunocompromised patients [4, 5]. There are cases when diseases with a severe clinical course (herpes pneumonia, meningoencephalitis, extensive mucocutaneous lesions, etc.), which can lead to the death of the patient, were associated with ACVresistant HSV variants [9-9].

HSV resistance to ACV is due to mutations in the* UL23 *gene encoding thymidine kinase (TK) and/or* UL30 *encoding DNA polymerase, with which is associated a mechanism of drug action. The first stage of ACV phosphorylation resulting in ACV monophosphate formation is catalyzed by viral TK, while the next two stages are catalyzed by host cell enzymes. ACV triphosphate (the active metabolite of ACV) inhibits viral DNA polymerase and, moreover, inhibits elongation through the mechanism of termination, being incorporated in the newly synthesized DNA chain [10]. Resistance of HSV clinical isolates to ACV is in 95% of the cases due to mutations in the *UL23 *gene, which, as a rule, lead to a loss or significant reduction in TK activity (96%). Mutants with altered substrate specificity to TK are much more rarely isolated (4% of all TK mutants) [11, 11].

It is important to note that due to the similarity between the mechanisms of ACV and PCV action, the resistance of HSV to these drugs has in most cases an overlapping profile, which leads to a reduction in the efficiency of the therapy using ACV, PCV or their metabolic precursors. Trisodium salt of phosphonoformic acid (PFA, foscarnet) is used in such cases in international practice [12, 13]. In addition, there is a positive experience of using CDV (cidofovir, Vistide) in the most severe cases when PFA also turned out to be ineffective [14]. These drugs are in most cases equally effective in inhibition of the reproduction of HSV variants both sensitive and resistant to ACV and PCV. Unfortunately, PFA and CDV are highly toxic and their use is prohibited in Russia. HSV mutants that share cross-resistance to ACV/PCV and PFA and/or CDV have been also described [15-17]. Therefore, the development of agents effective against HSV and preventing a recurrence of the disease is highly relevant.

Phosphite of acycloguanosine (Hp-ACG, phosphonate of 9-[(2-hydroxyethoxy)methyl]-guanine) is an H-phosphonate derivative of ACG. We have shown earlier in an HSV-1 model that Hp-ACG exhibits antiviral activity both *in vitro *(Vero E6 cell culture) and *in vivo *(i.p. administration in infected mice). The drug is active not only against the HSV-1/L_2_ reference strain, but also against the ACV-resistant laboratory strain HSV-1/L_2_/R and resistant clinical isolates of HSV-1 (Avd and Sha), which have epidemic value [18-21]. Thus, Hp-ACG may be of interest to practical medicine as a drug that effectively suppresses the infection caused by ACV-sensitive and -resistant variants of HSV-1.

In the present study, we have determined the efficiency of orally administered Hp-ACG in mice with a generalized herpes infection, as well as conducted a comparative study of the ointment formulation of Hp- ACG and ACV for the development of drugs for the treatment of a cutaneous herpes infection.

## EXPERIMENTAL SECTION

**Compounds**

Hp-ACG was synthesized at the V.A. Engelhardt Institute of Molecular Biology, RAS. ACV (9-[(2-hydroxyethoxy) methyl]-guanine) (GlaxoSmithKline, USA/UK) was used as a reference drug.

**Viruses and cells**

HSV-1/L_2_ strain from the State Collection of viruses at the D.I. Ivanovsky Institute of Virology was used in this study. A line of Vero E6 cells (African Green Monkey Kidney Cells) was kindly provided by A.M. Butenko (D.I. Ivanovsky Institute of Virology, Ministry of Healthcare of the Russian Federation).

**Animals**

Agouti guinea pigs of 250 g weight (males) and BALB/c white mice weighing 8–9 g (males) were obtained from the nursery Stolbovaya Branch of the Russian Academy of Medical Sciences (Moskovskaya oblast, Chekhovskiy rayon, Stolbovaya pos.). The animals were healthy, and they had a veterinary certificate of quality and health. All procedures were carried out strictly in full compliance with the requirements stated in Rules of Work with the Use of Experimental Animals № 755 dated 08/12/1977.

**Cytotoxicity**

Cytotoxicity was evaluated in accordance with the conventional trypan blue exclusion method of cell staining [20, 21]. CTD50 was considered to be the substance concentration which ensured the death of 50% the cells after 72 h of incubation.

**Antiviral activity**

The antiviral activity of the compounds *in vitro *was assessed using the micromethod by their ability to protect infected cells from death by preventing the development of a virus-induced CPE in accordance with the method designed by De Clercq E. *et al*. [22] as we have previously described [20, 21]. A monolayer Vero E6 cell culture grown in plastic 96-well plates (Linbro, Flow laboratories, UK) was infected at a multiplicity of 0.1 PFU/cell; the duration of incubation was 48 hours at 370C. In the control viral CPE was developed to 95– 100%: i.e., it covered the entire monolayer of cells. Efficacy of the agent was quantitatively expressed as ID50 and ID95, concentrations of the compound that inhibit the development of virus-induced CPE by 50% and almost completely, respectively.

**Antiherpetic activity**

Antiherpetic activity of the compounds was studied on an experimental model of mice with a generalized infection. HSV-1/L2 adapted to mouse brain was used. Infectious material was introduced i.p. in a volume of 200 μl at a dose provoking the death of 95 or 50% of the animals in the infected, untreated control group. The infecting dose is specified for each experiment separately. The animals received the studied compounds dissolved in thrice-distilled water or saline i.p. or orally in a volume of 0.2 ml. Single doses of the compounds and the number of animals in the group are specified separately for each experiment. The compounds were administered twice daily for 5 days, and the first administration was 1 h after infection. The observation period was 21 days. The effectiveness of the drugs was evaluated based on their ability to protect the animals from death (survival rate compared to infected, untreated control group) and an increase in the MDD. For a maximum MDD has been accepted as 21 days (observation period).

A total of 96 h after infection, when infectious titer reached a maximum value in the brain of the infected animals, three mice from each group were sacrificed. Brain and lungs were isolated and homogenized in Dounce homogenizer at a temperature of 40C, and a 10% suspension was prepared in saline, centrifuged at 5,000 rev/min and 40C for 10 min. Infectious titer of the virus was determined in the supernatant by plaque formation by titration in cell culture as described below. The efficacy of the drug was assessed by a reduction in infectious virus titer values in organ material from animals of the experimental group receiving the drug compared to the control (infected but not treated animals).

In order to assess drug efficacy in an experimental cutaneous infection [23], viral material was applied with its subsequent rubbing on the scarified after depilation of skin areas of the guinea pigs of about 5 cm^2^. Viral titer was 7.87 lg PFU/ml.

At 48 h after inoculation with HSV slight redness appeared on the infected surface , followed by treatment initiated with the application of ointment twice daily for 5 days. PEG 600 was used as a base for the preparation of the ointment.

**Infectious virus titer**

The value of infectious virus titer in vesicle fluid was determined by plaque formation assay as described below. Vesicular fluid intake was conducted 4 days after infection. Infectious virus titer was determined by plaque assay. The 24-hour culture of cells grown in 24-well plastic plates (Costar, USA) was infected with 10-fold dilutions of the virus. After 1 h, the cell monolayer was washed from non-adsorbed virus with saline, a coating medium (2 ml/well) was added, and the cells were incubated at 370C in 5% CO_2_ atmosphere. The overlay consisted of Eagle’s and 199 mediums (produced at the M.P. Chumakov Institute of Poliomyelitis and Viral Encephalitis RAMS, Russia) mixed at a ratio of 1 : 1 with 5% fetal bovine serum and 0.4% agarose (Sigma, USA). After 48 h, the overlay was removed, the infected cultures were fixed with 10% neutral formalin, stained with a 0.5% gentian violet solution, and the plaque number was counted [24].

Infectious titer T was calculated using the formula

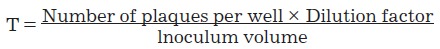



and expressed as lg PFU/ml, where PFU is the plaque forming unit.

## RESULTS AND DISCUSSION

We have shown that Hp-ACG is equally effective in the selective inhibition of the reproduction of both ACV-sensitive and -resistant HSV strains, including laboratory strain HSV-1/L2/R and the clinical isolates Avd and Sha, which circulate in the human population and now are among the most frequent TK-negative/ deficient phenotypes in clinical practice [18, 19]. The corresponding data are shown in *Table 1*.

**Table 1:** Antiviral activity of Hp-ACG and ACV in a Vero E6 cell culture [18, 19]

Compound	Characteristic	Virus			
		HSV-1/L_2_	HSV-1/L_2_/R	Avd	Sha
Hp-ACG	ID50, μg/ml	15.6	31.25	31.25	31.25
	ID95, μg/ml	31.25	62.5	250	62.5
	SI	> 64	> 32	> 32	> 32
ACV	ID50, μg/ml	0.39	> 400	3.9	12.5
	ID50, μg/ml	1.56	> 400	31.25	50
	SI	> 1026	> 1	> 102	> 32

Note. HSV-1/L2 – reference virus strain; HSV-1/L2/R – laboratory virus strain highly resistant to ACV; Avd and Sha – ACV-resistant clinical isolates of HSV-1. Multiplicity of infection – 0.1 PFU/cell. Duration of incubation – 48 h. The results of two independent experiments are presented. CTD50 for ACV and Hp-ACG equal > 400 and > 1000 μg/ml, respectively. SI was calculated as the ratio of CTD50 to ID50.

The results obtained *in vitro *were confirmed in experiments* in vivo *on a model of experimental HSV infection in BALB/c white mice. Since the infection caused by the ACV-resistant HSV-1/L2/R variant was not lethal, the efficiency of Hp-ACG in i.p. administration was evaluated based on the influence on viral accumulation in the brain of the animals. It was demonstrated that the introduction of Hp-ACG (450 mg/kg, twice per day) led to a decrease of virus titer of 1.30 lg PFU/ml (from 3.31 ± 0.16 to 2.01 ± 0.11) of day 4. This result was close to the effect of Hp-ACG in similar conditions on the model of HSV-1 reference strain: virus titer decreased from 5.49 ± 0.25 to 4.01 ± 0.16 lg (mortality in the control in the latter case was 92.5%, mortality decreased to 67.5% in the experimental group, and MDD increased from 4.90 ± 0.75 to 10.25 ± 1.20) [18].

We have established a mechanism of resistance formation in HSV-1/L2/R, Avd, and Sha strains. HSV-1/L_2_/R and Sha strains contain mutations in the* UL23 *gene, which lead to R220H substitution in TK and, as a result, to a loss of enzyme activity. Such an enzyme is incapable of catalyzing the first step of ACV phosphorylation required for its conversion into active metabolite ACV triphosphate, which is capable of incorporating into a newly synthesized viral DNA chain and aborting its elongation through the mechanism of termination. Deletion deltaT66 was found in the *UL23* gene of the Avd strain located in close vicinity to the ATP-binding site of the enzyme (amino acid residues 51–63), which may cause a decrease in ACV phosphorylation efficiency [20, 25]. The obtained results confirm that all the ACV-resistant HSV strains included in the study exhibit a TK-deficient phenotype and allow one to suggest that mutations in the nucleoside binding site and ATP-binding center do not substantially affect the metabolic conversions of Hp-ACG to monophosphate of ACV, which, like ACV, is further converted into diand triphosphate of ACV. However, this conversion likely occurs through an alternative pathway by passing the stage of degradation of Hp-ACG to ACV [21].

The development of herpes encephalitis is known to be the cause of death in animals with a generalized herpes infection, and in order to exhibit antiviral activity, the drug has to overcome the blood-brain barrier and enter the brain of the infected animals. Apparently, i.p. introduction of Hp-ACG results in a concentration of the drug into the animal brain that can effectively inhibit virus reproduction. However, the bioavailability of drugs upon *per os *administration is lower than in the case of i.p. or intravenous administration and the drug dosage should be increased in order to preserve its antiviral efficacy. For example, the bioavailability of ACV when administered orally does not exceed 10–20%. The maximum concentration of ACV in blood serum upon *per os *administration of 200 mg of the drug is 0.4–0.8 μg/ml. Intravenous administration of a standard dose (5 mg/kg, three times a day) provides a significantly greater concentration of ACV in serum; about 9.8 mg/ml [26]. In a series of cases, oral administration fails to be effective. For instance, because of the extremely low bioavailability of CDV upon *per os *administration ( < 5%), the drug is administered only intravenously [27]. The bioavailability of PCV upon oral administration is also very low: 5% [28]. In this regard, PCV is used only in the form of its metabolic precursor, famciclovir, for oral administration. Structural modifications allow one to increase the absolute bioavailability of PCV from famciclovir up to 77% after a single oral administration [29].

Therefore, having continued the logical development of the research we conducted on the antiherpetic activity of Hp-ACG *in vitro *and *in vivo *[18, 19], we studied the ability of Hp-ACG to protect mice with an experimental generalized infection caused by HSV-1 from death upon oral administration.

The pathogenicity of ACV-resistant HSV variants containing mutations in the TK gene is known to be defined by their phenotype, since it depends on the level of TK activity. Experiments on laboratory animals have shown that TK-negative and TK-deficient strains usually exhibit a substantially reduced virulence or are avirulent; i.e., incapable of causing lethal infection in laboratory animals and manifesting as zosteriform [30, 31]. As it has been noted, the ACV-resistant strains of HSV-1/L2/R, Avd and Sha used in a previous series of experiments belong specifically to the TK-negative or TK-deficient phenotypes: therefore, we further infected animals with an initial parent strain HSV-1/L2 with unchanged drug sensitivity. We would like to emphasize that the ability of TK-deficient and TK-negative strains to cause severe clinical forms of a herpes infection is caused by their natural heterogeneity. The presence of TK-positive virus particles capable of synthesizing functionally active TK in a population allows one to compensate for the lack or a low level of TK activity of ACV-resistant viral particles [32, 32].

The infected animals in experimental groups received Hp-ACG as a triple-distilled water solution as described in Experimental Section. Single doses of Hp- ACG were chosen based on the data on drug efficacy upon i.p. administration in the model of experimental infection in mice caused by HSV-1: the ability of Hp- ACG to protect infected animals from death was 3–4 times lower compared to ACV [18].

**Table 2:** Influence of orally administered Hp-ACG on the survival rate of BALB/c mice (8.41 ± 0.31 g) infected with HSV-1/L_2_

Compound	Route of administration and dose of the drug	No. of survivors/ total no. animals in the group	Mortality, %	Protection, %	MDD days	Yield of virus in brain, lg PFU/ml
–(virus control)	-	26/60	56.67 ± 3.33	-	11.35 ± 1.11	4.18 ± 0.18
Hp-ACG	300 mg/kg × 2 times daily/5 days	29/40	27.50 ± 2.50	29.17	16.40 ± 1.15	3.06 ± 0.12
Hp-ACG	400 mg/kg × 2 times daily/5 days	33/40	17.50 ± 2.50	39.17	18.13 ± 1.00	2.56 ± 0.05
Hp-ACG	500 mg/kg × 2 times daily/5 days	37/40	7.50 ± 2.50	49.17	19.78 ± 0.69	2.03 ± 0.15
ACV	100 mg/kg × 2 times daily/5 days	14/40	35 ± 0	21.67	15.05 ± 1.30	3.45 ± 0.05

Note. The infectious doseis 4×10^6^ PFU/mouse (titer of virus-containing material 7.30 lg PFU/ml). The results of two independent experiments are presented.

The results presented in *Table 2* demonstrate that Hp-ACG administered orally is effective in protecting animals from death under the proposed experimental conditions. The protective effect is of pronounced dosedependent nature: an increase in the Hp-ACG dose leads to increased survival rates and MDD of animals, which is due to the inhibition of viral replication in the brain of infected animals. However, the efficiency of Hp-ACG upon oral administration was lower than in the case of i.p. administration with almost equal animal mortality in the control groups. Thus, at 57% mortality in the control group, 29 out of 40 animals receiving the drug *per os *at a dosage of 300 mg/kg died. The efficacy of Hp-ACG upon i.p. administration at the same single dose and pattern was significantly higher: only one mouse died out of 40. Mortality in the control group in this case was 60% [19].

Reduction in efficiency of Hp-ACG and ACV upon oral administration (twice daily for 5 days) in comparison with i.p. introduction in the same manner was approximately the same in both cases: oral efficacy of ACV was 3–4 times lower than i.p. Thus, in accordance with our data, the protective effect of ACV upon i.p. administration at a single dose of 25 mg/kg was 30% and the increase in MDD was 5.2 days with 65% mortality in the control group [33], which is well comparable with the efficiency values of ACV administered in mice* per os *at a dose of 100 mg/kg (*Table 2*).

It is important to underline the fact that the efficacy of Hp-ACG upon oral administration at a single dose of 300 mg/kg is well comparable with ACV efficiency at a single dose of 100 mg/kg administered in the same manner and under the same experimental conditions. Protection of the animals from death in the latter case was 21.67%, the increase in MDD was 3.7 days, and the reduction in viral titer in the brain was 0.73 lg.

A comparison of these data with the results shown in *Table 1* indicates that the difference in the efficacy values of Hp-ACG and ACV in a generalized model of HSV infection was less significant than in *in vitro *experiments. It is possible that structural modification of the ACV molecule (incorporation of a H-phosphonate group) leads to a change in the pharmacokinetic parameters of the drug; for example, it increases its bioavailability upon oral administration.

The antiviral effect of Hp-ACG at a single dose of 500 mg/kg administered orally was close to the effect of Hp-ACG at a dose of 300 mg/kg upon i.p. introduction (three mice out of 40 died). The values of MDD and decrease in virus titer in the brain were also comparable (the difference in MDD from the control group was 9.64 and 8.43 days, and the decrease in virus titer was 3.47 and 2.15 lg PFU/ml upon i.p. and oral administration, respectively). Thus, we can conclude that the antiherpetic efficacy of Hp-ACG administered orally is reduced by less than 2-fold compared to i.p. administration. Since oral administration is more convenient and painless and does not require qualified medical personnel, the obtained results may be of practical interest.

The most common forms of herpes infection are lesions of the skin and mucosas, which do not affect internal organs and the CNS. For example, a recurrence of orofacial herpes (herpes *labialis*) is observed in 15–45% of the adult population [34]. Since herpetic lesions in most cases affect limited areas, the systemic administration of antiherpetic drugs may be counterproductive. Optimal dosage forms in this case are ointments, creams, and gels for topical application. In case of effective drug penetration through the skin, this method not only ensures a selective influence directly on the damaged tissues, but also provides therapeutic concentration of the antiviral agent at the site of the infection when used in much lower doses than upon systemic administration. Consequently, the toxic effect of the drug on the organism is lower, the risk of unwanted side effects is minimized, and, which is also important, the cost of the therapy is also lower. Therefore, we considered appropriate to examine the effectiveness of Hp-ACG in an ointment formulation in the next stage of the research.

Since an experimental cutaneous HSV infection in guinea pigs is caused by the same virus as in humans, and its manifestations are similar to the clinical course of a cutaneous HSV infection in humans, we used the same model for the examination of the compounds for antiherpetic activity.

The safety of skin applications of 5% and 10% Hp- ACG ointments and PEG 600, which served as the ointment base in our experiments, was first evaluated in intact guinea pigs. Ointments were applied on depilated areas of the skin twice daily for 5 days. There was no redness or ulceration of the skin at the site of ointment application, change in animal behavior, or loss of appetite.

In order to evaluate the efficacy of the ointment formulation of Hp-ACG, the animals were infected as previously described in detail in [35] and stated in Experimental section. We used the culture HSV-1/L_2_ virus, which is less neurovirulent than fresh clinical isolates, or a laboratory virus passaged through a mouse brain in order to prevent the development of a generalized infection leading to the death of the animal from encephalitis.

Since accurate calculation of the drug dosage for local use is not possible, the tested ointments were applied in a thin layer directly on to the affected skin areas. Placebo ointment, vehile consisting of PEG-600 without the drug, was applied in the control under the same conditions.

Visual assessment of the clinical manifestations of the experimental infection was evaluated daily. The formation of local lesions typical of a herpes infection was found in the control group on the 3rd day (72 hours after infection): bullous rash (vesicles) of 2 mm both grouped and single. After another 24 hours (day 4 of infection), the intensity of the lesions reached its maximum and then gradual drying of herpetic vesicles took place with crust formation. After 7–8 days of virus inoculation, the reverse process took place (crust rejection). On day 12, a complete reepithelialization was observed.

A significant reduction in the severity of the clinical symptoms of infection, as well as reduction in the time of treatment under the action of the studied substance in comparison with the control, was used for a quantitative characterization of the compound’s activity. The corresponding results are reported in *Table 3*.

**Table 3:** Comparison of the therapeutic effect of the ointment formulation of Hp-ACG and ACV an in experimental model of a cutaneous herpetic infection in guinea pigs caused by HSV-1/L_2_

Compound	Compound concentration in ointment, %	Total Lesion area		Number of herpetic sores		Virus titer in vesicular fluid, lg PFU/ml	Start of reverse process, days	Complete reepithelial- ization, days
		Smean, cm^2^	Decrease compared to control, %	n_mean_	Decrease compared to control, %			
– (control)	0	4.62 ± 0.08	-	11.25 ± 0.49	-	4.11 ± 0.10 (3.78–4.54)	7-8	12
Hp-ACG	5	4.36 ± 0.07	5.76	10.75 ± 0.31	4.44	4.00 ± 0.08 (3.60–4.38)	6	11
	10	4.22 ± 0.07	8.60	10.00 ± 0.27	11.11	3.85 ± 0.11 (3.54–4.40)	6	11
ACV	5	4.30 ± 0.18	6.95	10.00 ± 0.38	11.11	3.89 ± 0.15 (3.40–4.48)	6	11

Note.The ointment was applied twice daily for 5 days. The first application was 48 hours after infection when a slight redness appeared. The results were registered 4 days after infection. Virus titer in vesicular fluid was determined after 4 days of inoculation when the clinical severity of herpetic manifestations reached their maximum value in the control. The results of two independent experiments are presented.

In the case of usage of an ointment of Hp-ACG (5%), the average area of lesions in the experiment was 5.76% lower than in the control (P < 0.05 when evaluated using Student’s t-test) after 2 days (4 days after virus inoculation), while the number of herpetic sores was 95.55% compared to the control (P < 0.3). The term of the start of the reverse process and cure (complete reepithelialization) was decreased by 1 day compared to the control when using a 5% Hp-ACG ointment.

The therapeutic effect of Hp-ACG was more pronounced when used as a 10% ointment and also well comparable to the effect of a 5% ACV ointment. A milder clinical course of infection was observed in both cases during the entire period of observation, which was expressed as a decrease in the number of vesicular structures and reduced lesion surface area. The difference between the values obtained in the experimental and control group was statistically significant (P < 0.05 using Student’s t-test). The reverse process and cure of the guinea pigs occurred 1 day earlier than in the control group.

The data presented in *Table 3* demonstrate that, in all cases, the use of an ointment pharmaceutical form did not lead to a significant reduction in infectious virus titer in vesicular fluid. Therefore, it would be wrong to assume that the ability of Hp-ACG to inhibit the development of a cutaneous herpes infection in guinea pigs caused by a HSV-1/L2 reference strain is due to the inhibition of viral replication at the site of the infection. However, the obtained results are in good agreement with the published data on the influence of ACV upon local administration on infectious titer of HSV under similar experimental conditions. At the same time, a statistically significant decrease in the number of herpetic vesicular formations and a reduction in the lesion surface area were noted [36-38]. Apparently, the effect of Hp-ACG upon local administration has a primarily preventive character, which is expressed in a prevention of the formation of herpetic sores upon drug application during the prodromal phase at the stage of the appearance of a slight redness but not in the inhibition of virus replication in already-formed vesicles.

## CONCLUSION

Thus, our study of the therapeutic efficacy of Hp-ACG action in *in vivo *experiments revealed that Hp-ACG effectively influences a generalized and cutaneous infection caused by HSV-1upon *per os *administration or as an ointment pharmaceutical form. Despite the fact that this compound is inferior to ACV (the concentration of Hp-ACG must be increased 2-fold in order to achieve a comparable therapeutic effect), it can inhibit the reproduction of ACV-resistant virus variants, as we have shown in experiments *in vitro *and *in vivo*. This makes possible the use of Hp-ACG when ACV is no longer effective.

## Abbreviations

HSV-1 and HSV-2: Herpes Simplex virus type 1 and type 2, respectively
TK: thymidine kinase;
ACV/ACG: acyclovir/acycloguanosine
Hp-ACG: phosphite of acycloguanosine
PCV: penciclovir;
PEG 600: polyethylene glycol 600
MDD: median day of death
PFA: phosphonoformic acid
CDV: cidofovir
CTD50: 50% cytotoxic dose
CPE: cytopathic effect
ID50 and ID95: 50% and 95% inhibitory dose
SI: selectivity index
i.p.: intraperitoneal
